# Variation of vitamin B contents in maize inbred lines: Potential genetic resources for biofortification

**DOI:** 10.3389/fnut.2022.1029119

**Published:** 2022-10-21

**Authors:** Fardous Mohammad Safiul Azam, Tong Lian, Qiuju Liang, Weixuan Wang, Chunyi Zhang, Ling Jiang

**Affiliations:** ^1^Biotechnology Research Institute, Chinese Academy of Agricultural Sciences, Beijing, China; ^2^Sanya Institute, Hainan Academy of Agricultural Sciences, Sanya, China

**Keywords:** *Zea mays*, thiamine, riboflavin, niacinamide, pantothenic acid, pyridoxine, variations, biofortification

## Abstract

Vitamin B and its derivatives possess diverse physiological functions and are essential micronutrients for humans. Their variation in crops is important for the identification of genetic resources used to develop new varieties with enhanced vitamin B. In this research, remarkable variations were observed in kernels of 156 maize inbred lines, ranging from 107.61 to 2654.54 μg per 100 g for vitamin B1, 1.19–37.37 μg per 100 g for B2, 19.60–213.75 μg per 100 g for B3, 43.47–590.86 μg per 100 g for B5, and 138.59–1065.11 μg per 100 g for B6. Growing inbreeds in Hainan and Hebei provinces of China revealed environmental and genotype interactions among these vitamins and the correlations between them in maize grain. Several inbred lines were identified as good sources of vitamin B and promising germplasms for maize breeding, namely By855 and Si273 are overall rich in all the studied vitamins, and GY386B and CML118 are specially enriched with derivatives of vitamin B6. The present study can assist maize breeders with germplasm resources of vitamin B for biofortification to offer people nutritious foods.

## Introduction

Vitamin B, a class of water-soluble vitamins, plays essential physiological roles in human health and must be obtained externally from foods (1). From the epidemiological perspective, deficiencies of vitamin B, including thiamine (vitamin B1), riboflavin (vitamin B2), niacin (vitamin B3), pantothenic acid (vitamin B5), pyridoxine (vitamin B6), folate (vitamin B9), cobalamin (vitamin B12) and their derivatives, have major outcomes in human health around the world. Such as, adverse pregnancy [B9 and B12, (2)], improper neonatal development [B9, (3)], peripheral neuropathy [B1, B6, and B12; (4)], limited brain development [B2, (5); B5, (6)] and reduced mental health [B1, (7); B3, (8); B6, (9)] are well-studied. Inadequate consumption of these vitamins causes hidden hunger, a kind of micronutrient malnutrition that is prevalent in populations of low-income countries (10). Along with these, the COVID-19 pandemic has unprecedentedly emerged as a global crisis in low and middle-income countries’ ability to afford and increase the consumption of healthy and nutritious food among the population (11). Under these circumstances, it is suggested to accelerate the development of accessible biofortified and post-harvest fortified food sources to fight against the hidden hunger (11). Extensive studies have been carried out on plants, such as barley [*Hordeum vulgare*, tocotrienols, (12)], cassava [*Manihot esculenta*, vitamin B1, (13); vitamin B6, (14)], maize [*Zea mays*, β-carotene, (15)], potato [*Solanum tuberosum*, folate, (16)], tomato [*Solanum lycopersicum*, folate, (17)], rice [*Oryza sativa*, folate, (18); vitamin B6, (19)], and wheat [*Triticum aestivum*, provitamin A, (20)], for modification of vitamin metabolism, with a goal to develop new varieties with elevated vitamin levels, thus offering people with nutritious foods.

Maize (*Zea mays* L.) is the second most abundant cereal crop, trailing only rice, and it occupies 160 million hectares of production area globally (21, 22). The edible part is the maize kernel, which has been chosen as a staple food by large populations and provides immense dietary support for them in Asia, Africa, and Latin America (23). Moreover, the crop is termed as “corn” in America and India, where this terminology means “that which sustains life” (24). The grains of maize are relatively rich in vitamins, antioxidants, bioactive compounds, and minerals (25, 26). In particular, it is a potential source of carotenoids, phenolic compounds and phytosterols (27–29). It’s reported that contents of vitamin B1, B2, B3, B5, and B6 in common maize grown in America (30) were far away to meet the recommended dietary allowance (RDA) for each vitamin B [1.2 mg/day for vitamin B1, 1.3 mg/day for B2, 16 mg/day for B3, 5 mg/day for B5, and 1.3 mg/day for B6; (31)]. Maize biofortification for provitamin A, vitamin B9, and vitamin E has been studied by Blancquaert et al. (18). Successful progress was gained in similar strategies for hybrids through molecular breeding after the discovery of the *opaque2* gene to enrich maize with vitamins by Das et al. (32); vitamin E, provitamin A, lysine and tryptophan, (33); tryptophan, (34); provitamin A, lysine, and tryptophan, and (35); provitamin A, lysine and tryptophan. However, seldom reported on other vitamin B in cereals, specifically in maize.

Nutritious crops can be developed by enhancing vitamin B via genetic breeding. Maize inbred lines are important genetic resources in the quest to elucidate genetic relationships (36), breed desirable traits (37) and enhance the quality of germplasm (26). Even though a study on nutritional composition including macronutrients, minerals, and antioxidants was conducted on Indian maize germplasms by Langyan et al. (38), reports on detailed investigation of vitamin B contents in different maize germplasms are rarely available. Thus, if the contents and variations of vitamin B in large amounts of inbred lines are measured across locations, several prospective lines with high levels of vitamin B can be identified and useful for biofortification and relevant research on vitamin B metabolism.

In this study, 156 maize inbred lines and 32 commercial hybrid varieties were comprehensively profiled and investigated for vitamin B1, B2, B3, B5, and B6 in the kernels using liquid chromatography-tandem mass spectrometry (LC/MS-MS). The results can facilitate the identification of the vitamin B-rich-maize genetic resources for a breeding program, thus providing nutritious foods for human health improvement.

## Materials and methods

### Materials

A total of 156 maize inbred lines were grown in Ledong, Hainan, China (longitude: 108.884767; latitude: 18.708674) in the spring of 2018, and samples of the dry seed powder for vitamin profiling were kindly provided by Yan (39). The same 156 inbred lines, together with 32 commercial hybrid varieties collected from Northern China, were grown in Langfang, Hebei, China (longitude: 116.61489; latitude: 39.608688) in the summer of 2018. These trials involved a 13 × 12 alpha lattice design with three replications. The experimental field was loamy soil with pH 6.8, organic matter 0.7%, phosphorus 13.8 mg/L, and potassium 48 mg/kg. During field preparation, 440 kg/acre of urea (46-0-0) was applied. The herbicides were applied 5 days after planting. Three seeds were hand planted in a hill and thinned to one plant after emergence, where planting remained as 5 m long rows with row and planting spacing of 60 and 25 cm, respectively. Five ears from each inbred line were harvested and air-dried. Kernels from the middle part of each ear were mixed and used for vitamin B analysis.

### Chemicals

Reference standards of thiamine hydrochloride (vitamin B1); riboflavin (vitamin B2), niacinamide (vitamin B3), D-pantothenic acid hemicalcium salt (vitamin B5), pyridoxal hydrochloride (PL), pyridoxal-5’-phosphate hydrate (PLP), pyridoxamine dihydrochloride (PM), pyridoxamine-5’-phosphate (PMP), pyridoxine hydrochloride (PN), were obtained from Sigma Chemicals (Sigma USA).^1^ All other chemicals and reagents used in this study were purchased from Solarbio (Beijing China),^2^ including sodium hydroxide, high-performance liquid chromatography (HPLC)-grade acetonitrile, phosphoric acid, and methanol. Ultra-pure water was supplied by a Milli-Q water purifier system from Millipore (Merk German).^3^

### Stock and working solutions

Stock solutions of reference compounds (vitamin B1, vitamin B3, vitamin B5, PL, PLP, PM, PMP, PN) comprised 1 mg/ml diluted with ultra-pure water. Next, 0.1% (V/V) sodium hydroxide was added to the stock solutions of vitamin B2 and PLP; 20 μl of the above stock solutions, excluding D-pantothenic acid hemicalcium salt (for which 40 μl was required), were diluted with ultra-pure water into mixed standard reserve solutions of 10 μg/ml (10,000 ng/ml). All stock solutions and mixed standard reserve solutions were stored at –80^°^C until use.

All working standard solutions for calibration curves were prepared by diluting a mixed standard reserve solution with the extraction solutions at eight different concentrations (0.1–200 ng/mL). The linear standard calibration curves (*r*^2^ > 0.999) of vitamin B1, B2, B3, B5, and B6 were generated by injecting 0.1–200 ng/mL of standard solutions into 20 μL. All working solutions were stored at 4^°^C before use.

### Vitamin extraction and measurement by liquid chromatography-tandem mass spectrometry

All vitamin B was extracted according to the method with minimal changes of Jin et al. (40) and Truica et al. (41). One hundred seeds of each inbred line were grounded to be powder using SPEX Geno Grinder 2010 (SPEX SamplePrep, USA).^4^ All the powder was sealed in the plastic bags and stored in dark until the use. Briefly, vitamins were released from maize kernel samples (0.05 g) by adding 1 mL of 10% methanol + 0.1% phosphoric acid aqueous solution, vortex mixing for 20 s, and placing in a water bath at 100°C for 20 min. After cooling, samples were centrifuged at 13,000 rpm for 15 min. Supernatants were filtered using a polyether sulfone membrane. The filtered solution was separated on a TSK-GEL Amide-80 (4.6 × 100 mm, 5 μm; TOSOH Japan)^5^ using an HPLC instrument (Waters USA)^6^ equipped with a 5500 QTRAP mass spectrometer together with electrospray ionization (ESI) source (Applied Biosystems SCIEX USA).^7^. Elution was performed using a binary gradient of methanol:acetonitrile (65:35) (mobile phase A) and 10 mM ammonium acetate aqueous solution (containing 0.4% formic acid) (mobile phase B), according to the following program: 0 min, 40% A/60% B; 3.5 min, 85% A/15% B; 3.51 min, 40% A/60% B; 6.0 min, 40% A/60% B. The flow rate was 0.8 mL/min and the column temperature was 4°C. Analyte peak areas were normalized to standard peak areas and converted to nmol/L using the standard curve. The column temperature was maintained at 4^°^C. The injection volume was 20 μL for each experiment.

A Waters triple quadrupole tandem MS coupled with an ESI interface was used for mass analyses and quantification of target analytes using a modified method described by Zafra-Gómez et al. (42). The mass spectrometer was operated in positive-ion mode. The parameters were optimized for target analytes with a gas temperature of 500°C, nebulizer pressure of 50 psi, and capillary voltage of 5,500 V(+). The parameters for standards were m/z 265–122.1, 19 eV for vitamin B1, m/z 377.1–243, 39 eV for vitamin B2, m/z 123.1–80, 27 eV for vitamin B3, m/z 220–184, 19 eV for vitamin B5, m/z 170.1–151.9, 20 eV for PN, m/z 168.1–150, 18 eV for PL, m/z 169–152.2, 19 eV for PM, m/z 249.2–232, 20 eV for PMP, and m/z 248.1–150.2, 46 eV for the PLP, respectively. System operation, data acquisition, and data analyses were performed with Analyst 1.6.3 Quantitative Processing Software (Applied Biosystems, USA) and Microsoft Excel software (Microsoft, USA). The sum of the contents of PN, PL, PM, PMP, and PLP represents the total B6 level. Four technological replicates were used for the detection of each maize sample for each location.

### Statistical analysis

The contents of vitamin B in the different maize lines are shown as the mean with a standard deviation of all biological replicates, in micrograms per 100 g of grain. To determine differences in vitamin content between the two environments, the data was subjected to a two-sample independent *t*-test at a *P* < 0.05 (with a 5% significance level) using SPSS (version 26) statistical software for the two locations. We also conducted a two-way ANOVA to examine the significant differences in vitamin B content between two locations across the genotypes, using R version 4.2.0. Using R statistical software, the packages used were: PerformanceAnalytics, dplyr, and psych to examine Pearson’s correlation and draw scatter plots; pheatmap and dplyr to generate heatmaps; and FactoMineR and factoextra for principal component analysis (PCA). Furthermore, violin-box plots of the vitamins were visualized using OriginPro 2019b.

## Results

### Overall variations of vitamin B in maize inbred lines

Vitamin B contents significantly varied among the 156 maize inbred lines. The variations ranged from 107.61 ± 16.26 to 2654.54 ± 113.7 μg/100 g for vitamin B1, 1.19 ± 0.19 to 37.37 ± 2.44 μg/100 g for vitamin B2, 19.6 ± 0.39 to 213.75 ± 17.68 μg/100 g for vitamin B3, 43.47 ± 5.42 to 590.86 ± 4.18 μg/100 g for vitamin B5 (Supplementary Table 1). Total vitamin B6 is the sum of five derivatives (pyridoxal hydrochloride, PL; pyridoxal-5’-phosphate hydrate, PLP; pyridoxamine dihydrochloride, PM; pyridoxamine-5’-phosphate, PMP; pyridoxine hydrochloride, PN), and it ranged from 138.59 ± 12.08 to 1065.11 ± 56.93 μg/100 g (Supplementary Table 1). These 156 maize lines can be classified into four subgroups based on population structure: stiff stalk (SS) with 7 lines, non-stiff stalk (NSS) with 53 lines, tropical-subtropical (TST) with 47 lines, and a mixed (MIXED) group with 49 lines (39). However, less variation was found between the sub-groups for each vitamin (Supplementary Figure 1).

### Variations in vitamin B contents among the maize inbred lines grown in two locations

From the mean comparison performed among the inbred lines for each vitamin for both locations, a 9-fold- and a 24-fold-variation were observed for vitamin B1 in the grains from Hainan and Hebei, respectively (Supplementary Table 2). The mean content of Hebei grains was 1.44-fold of Hainan grains (854.28 ± 26.01 μg/100 g vs. 591.77 ± 11.62 μg/100 g; Table 1).

**TABLE 1:** Mean comparisons of vitamin B1, B2, B3, B5, and total B6, and the derivatives of B6 contents among inbred lines of Hainan and Hebei grain.

Location	Compound	Mean ± SD (μ g/100 g)	Compound	Mean ± SD (μ g/100 g)
Hebei	Vitamin B1	854.28 ± 26.01^a^	Pyridoxine	7.06 ± 0.22^a^
Hainan		591.77 ± 11.62^b^		4.67 ± 0.17^b^
Hebei	Vitamin B2	17.47 ± 0.34^a^	Pyridoxal	64.62 ± 1.31^a^
Hainan		5.84 ± 0.21^b^		45.67 ± 1.08^b^
Hebei	Vitamin B3	112.80 ± 1.86^a^	Pyridoxamine	231.65 ± 5.74^a^
Hainan		44.66 ± 0.77^b^		186.79 ± 4.80^b^
Hebei	Vitamin B5	293.30 ± 5.38^a^	Pyridoxamine-5’-phosphate	45.90 ± 1.12^a^
Hainan		232.36 ± 4.50^b^		60.67 ± 1.43^b^
Hebei	Total vitamin B6	403.44 ± 7.63^a^	Pyridoxal 5’-phosphate	54.21 ± 3.70^a^
Hainan		360.33 ± 6.07^b^		62.53 ± 2.55^a^

The data here are represented as means ± SD; letters (a and b) indicate significant differences among the pair of means in each row at the 1% level of significance. No significant difference is indicated when the values are followed by the same letter.

Similar trends were observed for vitamin B2, vitamin B3, vitamin B5, and total vitamin B6. A 22-fold and a 28-fold-variation were observed for vitamin B2, a 22- fold-, and a 9-fold-variation were observed for vitamin B3, an 8- fold-, and a 13-fold-variation were observed for vitamin B5, a 6- fold-, and a 7-fold-variation were observed for total vitamin B6, in the grains from Hainan and Hebei, respectively (Supplementary Table 2). The Hebei grains were 2.99 folds of Hainan grains for vitamin B2 (17.47 ± 0.34 μg/100 g vs. 5.84 ± 0.21 μg/100 g), 2.53 folds for vitamin B3 (112.80 ± 1.86 μg/100 g vs. 44.66 ± 0.76 μg/100 g), 1.25 folds for vitamin B5 (293.30 ± 5.38 μg/100 g vs. 232.36 ± 4.50 μg/100 g), and 1.45 folds for total vitamin B6 (403.44 ± 7.63 μg/100 g vs. 360.33 ± 6.07μg/100 g), respectively (Table 1). Similar to other vitamins B, PN, PL and PM were higher in Hebei grains than in Hainan grains, corresponding to 1.51 (7.06 ± 0.22 μg/100 g vs. 4.67 ± 0.17 μg/100 g), 1.41 (64.62 ± 1.31 μg/100 g vs. 45.67 ± 1.08 μg/100 g) and 1.24 (231.65 ± 5.74 μg/100 g vs. 186.67 ± 4.80 μg/100 g) fold, respectively. PMP and PLP were higher in the Hainan grains than in Hebei grains, but the difference in PLP between the two locations was not significant (Table 1).

In Hebei, various levels of variations were observed among the 32 commercial hybrid varieties, with vitamin B1 ranging from 145.78 μg/100 g to 769.14 μg/100 g, vitamin B2 from 11.14 μg/100 g to 106.27μg/100 g, vitamin B3 from 48.28 μg/100 g to 192.95 μg/100 g, vitamin B5 from 132.59 μg/100 g to 638.89 μg/100 g, and total vitamin B6 from 115.71 μg/100 g to 414.57 μg/100 g (Supplementary Table 2). Unlike the inbred lines, the hybrids did not show a significant difference with inbred lines from Hebei in contents of vitamin B2 and B3 as well as PN and PL. Meanwhile, the mean values of vitamin B1, B5 and B6 in the inbred lines were 2.52 (854.28 ± 36.59 μg/100 g vs. 338.54 ± 22.12 μg/100 g), 1.23 (293.30 ± 7.48 μg/100 g vs. 238.26 ± 16.53 μg/100 g) and 1.63 (403.44 ± 10.81 μg/100 g vs. 247.66 ± 12.84 μg/100 g) folds of that in the hybrids, respectively (Table 2).

**TABLE 2:** Mean comparisons of vitamin B1, B2, B3, B5, and total B6, and the derivatives of B6 contents between inbred and commercial hybrid lines in Hebei.

Location	Compound	Mean ± SD (μ g/100 g)	Compound	Mean ± SD (μ g/100 g)
Inbred	Vitamin B1	854.28 ± 36.59^a^	Pyridoxine	7.06 ± 0.31^a^
Hybrid		338.54 ± 22.12^b^		6.78 ± 1.11^a^
Inbred	Vitamin B2	17.47 ± 0.46^a^	Pyridoxal	64.61 ± 1.83^a^
Hybrid		20.8 7 ± 2.81^a^		72.52 ± 2.93^a^
Inbred	Vitamin B3	112.80 ± 2.56^a^	Pyridoxamine	231.65 ± 8.07^a^
Hybrid		98.06 ± 5.27^a^		121.65 ± 7.77^b^
Inbred	Vitamin B5	293.30 ± 7.48^a^	Pyridoxamine-5’-phosphate	45.90 ± 1.58^a^
Hybrid		238.26 ± 16.53^b^		23.71 ± 2.07^b^
Inbred	Total vitamin B6	403.44 ± 10.81^a^	Pyridoxal 5’-phosphate	54.20 ± 5.23^a^
Hybrid		247.66 ± 12.84^b^		23.00 ± 4.42^b^

The data here are represented as means ± SD; letters (a and b) indicate significant differences among the pair of means in each row at the 1% level of significance. No significant difference is indicated when the values are followed by the same letter.

### Variation in vitamin B accumulation as a response to the environment

**FIGURE 1:**
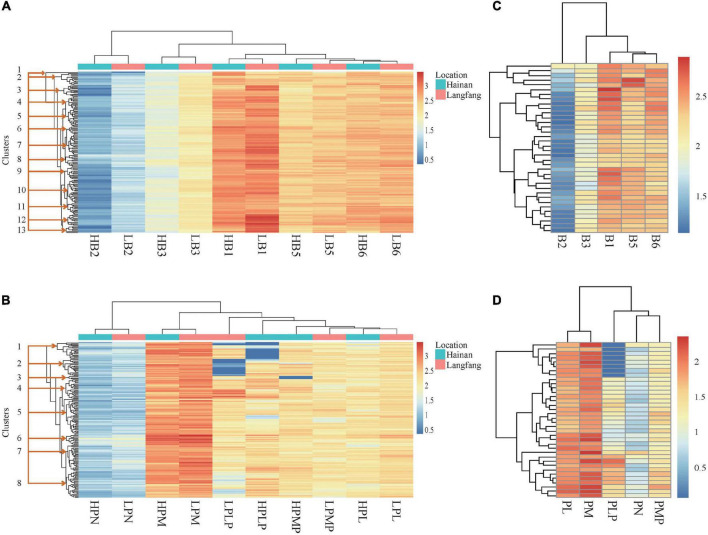
Heatmaps. **(A)** Vitamin B1, B2, B3, B5, and total B6 in Hainan and Hebei grain. **(B)** Derivatives of vitamin B6 in Hainan and Hebei grain. **(C)** Vitamins B1, B2, B3, B5, and total B6 contents of Hebei hybrids. **(D)** Derivatives of vitamin B6 in Hebei hybrids. Axes: **(bottom)**: variables; **(top)**: hierarchical clusters for closeness between variables; **(left)**: hierarchical clustering among lines. H, Hainan; L, Langfang (Hebei); B1, thiamine; B2, riboflavin; B3, niacinamide; B5, pantothenic acid; and B6, total of pyridoxine and derivatives; PL, pyridoxal; PLP, pyridoxal 5’-phosphate; PM, pyridoxamine; PMP, pyridoxamine-5’-phosphate; PN, pyridoxine.

To understand the effect of the environment across the two locations on vitamin B contents, the variation pattern was visualized in both heatmap and violin-box plot. The heatmap constructed using the Euclidean hierarchical clustered analysis (HCA) for vitamin B1, B2, B3, B5 and total B6, displayed 13 major clusters with distinguishable patterns (Figure 1A). Clusters 2, 3, 4, 7, 8, and 12 showed relatively high vitamin levels. Notably, clusters 3, 7, 8, and 12 and their sub-clusters consisted of the lines with high levels of vitamin B1 and B6. Genotypes with higher vitamin levels in these groups include Gy462, CML115 and P178 in cluster 3; M165, CIMBL139, Dan340, and B111 in cluster 7; CML113, M017, CIMBL96, GEMS33, By4960, EN25, and Gy1032 in cluster 8; GEMS61, Si273, CIMBL60, Gy386, GY386B, By855, and CIMBL146 in cluster 12. Additionally, the contents of vitamin B1 in Hebei grains in clusters 3, 7, and 13 were higher than those in Hainan grains (Figure 1A).

The heatmap plot generated eight major clusters for different vitamin B6 derivatives; however, the variation between the two locations was not prominent based on the coloring of Z-scores. Among the eight major clusters, the content of each derivative was moderately high in clusters 4, 6, and 7 (Figure 1B). The lines found to be high in vitamins are 4F1, W138, Gy1038, Si273 and Sy999 in cluster 4; By855, CIMBL60 and SW92E114 in cluster 6; CML170 and 835a in cluster 7. Similarly, the heatmap analysis highlighted that the variation range of the individual vitamin B6 derivatives was much larger among inbred lines than among the hybrid lines based on the Z-scores (Figure 1C), even though the hybrid lines were notably high in PM and PL (Figure 1D).

The distribution patterns and total ranges (25–75% within 1.5 IQR) of the vitamins were visualized using violin-box plots. We compared the distribution of the vitamins between inbred lines from Hainan and Hebei, and between inbred and hybrid lines from Hebei (Supplementary Figure 2). The vitamin B1 and B3 distributions in the inbred lines ranged twice as wider in Hebei than in Hainan; however, hybrid lines showed less variation (Supplementary Figures 2A,C). A skewed distribution was observed in both locations for vitamin B2 in the inbred lines, implying that the environment had a mild impact on this metabolite, whereas hybrid lines showed a wide range of variation (Supplementary Figure 2B). In the case of vitamin B5 and total B6, even though both locations showed skewed patterns of plots, the content range was wider in Hebei grains than in Hainan grains (Supplementary Figures 2D,E). Based on the distribution patterns, PL, PM, and PLP were found to be most affected by the environment (Supplementary Figures 2G,H,J). For instance, PM and PLP contents were nearly 1.5 and 2 folds wider in the range from the Hebei grains than the Hainan grains (Supplementary Figures 2H,J).

### Correlation among the vitamins

**FIGURE 2:**
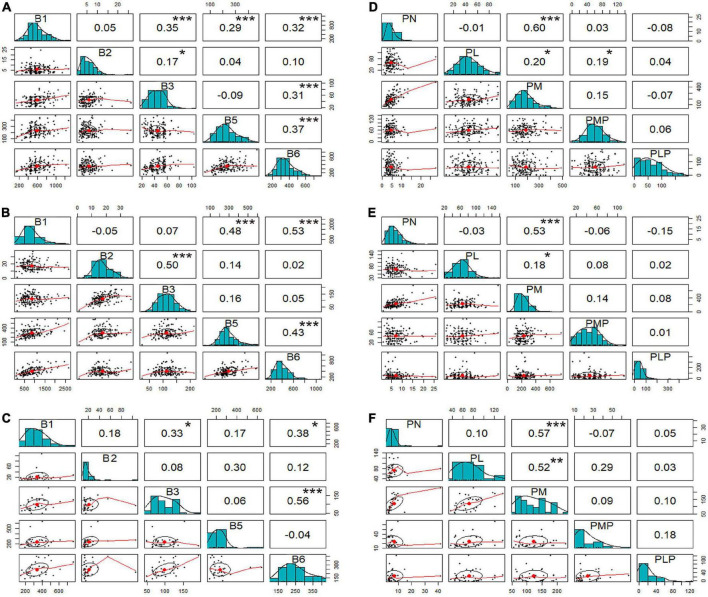
Scatter plots and correlation analysis among vitamin B. For each plot, the upper diagonal represents the correlation coefficient; *, **, and *** represent significance at *p* < 0.05, 0.01, and 0.001, respectively. Lower diagonal plots show the vitamin distribution. Plots: **(A)** vitamin B1, B2, B3, B5, and total B6 in Hainan; **(B)** vitamin B1, B2, B3, B5, and total B6 in Hebei; **(C)** vitamin B1, B2, B3, B5, and total B6 of hybrids; **(D)** derivatives of B6 in Hainan; **(E)** derivatives of B6 in Hebei; **(F)** derivatives of B6 in hybrids. B1, thiamine; B2, riboflavin; B3, niacinamide; B5, pantothenic acid; and B6, total of pyridoxine and derivatives; PL, pyridoxal; PLP, pyridoxal 5’-phosphate; PM, pyridoxamine; PMP, pyridoxamine-5’-phosphate; PN, pyridoxine.

Previous studies have demonstrated that some of the vitamins are metabolically connected (43). Hence, Pearson’s correlation analysis was performed in this study to examine relationships among the individual vitamin B based on the mean values of the inbred lines. The results obtained from the inbred lines grown in Hainan showed that vitamin B1 had a weak positive, but significant, correlation with vitamin B3, B5 and B6 (*r* = 0.35***, *r* = 0.29***, and *r* = 0.32***, respectively; Figure 2A). There were also significant and weak positive correlations between vitamin B6 and B3 (*r* = 0.31***) and B6 and B5 (*r* = 0.37***). Similarly, the inbred lines grown in Hebei showed moderately positive correlations of vitamin B1 with B3 (*r* = 0.50***), B5 (*r* = 0.48***), and B6 (*r* = 0.53***) (Figure 2B). There were also weak positive and significant correlations between vitamin B5 and B6 (*r* = 0.43***). The Hebei hybrids showed a moderate and positive correlation between vitamin B3 and B6 (*r* = 0.56***), similar to that of the inbred lines from Hainan (Figure 2C). For both locations, positive moderate and significant correlations were observed between vitamin B1 and B5, B1 and B6, and B5 and B6.

Separate scattered matrix plots were constructed and analyzed to identify correlations among individual derivatives of vitamin B6. It was observed that only PN showed a significant and strong positive correlation with PM, with the *r*-value being 0.60*** for the inbred lines from Hainan (Figure 2D), 0.53*** for the inbred lines from Hebei (Figure 2E), and 0.57*** for the hybrids (Figure 2F).

### Correlation of vitamins with maize genotypes

**FIGURE 3:**
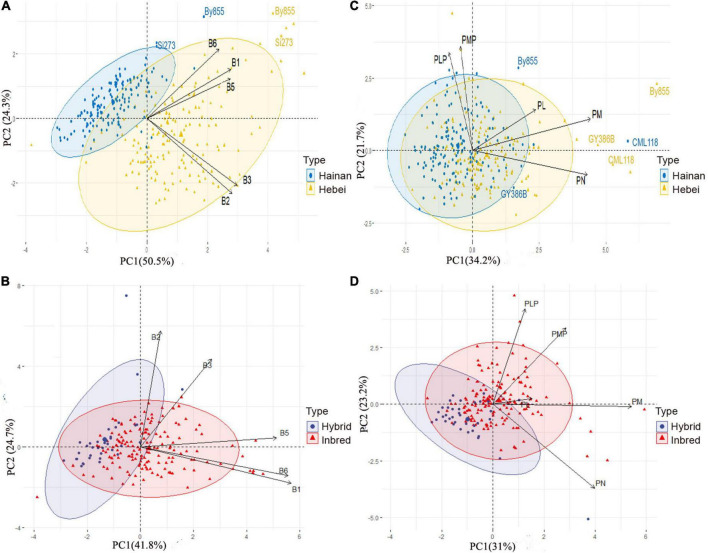
Principal component analysis (PCA) of biplots for vitamin B contents of maize inbred lines: **(A)** Vitamin B1, B2, B3, B5, and total B6 between Hainan and Hebei. **(B)** Vitamin B1, B2, B3, B5, and total B6 between inbred and hybrid. **(C)** Derivatives of vitamin B6 between Hainan and Hebei. **(D)** Derivatives of vitamin B6 between inbred and hybrid lines. PC1 as x-axis; PC2 as y-axis. B1, thiamine; B2, riboflavin; B3, niacinamide; B5, pantothenic acid; and B6, total of pyridoxine and derivatives; PL, pyridoxal; PLP, pyridoxal 5’-phosphate; PM, pyridoxamine; PMP, pyridoxamine-5’-phosphate; PN, pyridoxine. Biplots were generated through scattering the germplasms based on location with the content of vitamins indicated as vectors and the relative proportions of variability in each variable are indicated by the relative lengths of specific vectors.

Principal component analysis (PCA) was performed and presented in two-dimensional biplots to examine the relationships between observations (genotypes) and variables (vitamin B content), and among the variables as vectors. Two groups of maize lines, i.e., inbred lines grown in Hainan and Hebei, and hybrids and inbred lines grown in Hebei, were classed and used to obtain clusters in the plots (Figure 3). This method was used to identify the differences among lines from different groups and the major contributing variables and to investigate the interrelationships and co-linearity between the genotypes and vitamin B contents in two distinct eco-regions in China.

Most of the variations (74.8%) between the inbred lines from Hainan and that from Hebei were attributable to PC1 (50.5%) and PC2 (24.3%) for vitamin B1, B2, B3, B5, and total B6 (Figure 3A). The samples were classified into two distinguishable clusters in the biplot, showing location-dependent variation in vitamin B content. The Hebei genotypes were more diverse and scattered within the cluster because of their higher levels of vitamins compared to the Hainan genotypes. Vitamin B2 and B3 were closely associated and acted as the most important contributors to PC1. Meanwhile, vitamin B1 showed a close correlation with vitamin B6 and made the highest contribution to PC2. Among all the genotypes, cv. By855 and Si273 stood out from each cluster in the biplot with high levels of vitamins (Figure 3A). Likewise, a close positive correlation between vitamin B1 and B6 was observed for the inbred and hybrid lines grown in Hebei, and they largely contributed to PC1. In addition, vitamin B2 and B3 showed a close association and contributed to PC2 (Figure 3B). In this analysis, PC1 and PC2 accounted for 41.8 and 24.7%, respectively, of the total variation.

The overall variation in the B6 derivatives was analyzed based on the distributions and correlations between the measured variables, including PL, PLP, PM, PMP, and PN. In the corresponding biplot, PC1 was 34.2% and PC2 was 21.7%, which cumulatively comprised 55.9% of the total variation for the B6 derivatives, and PN was found to be the only contributor for PC1 (Figure 3C). The samples formed two overlapping clusters (one for each location) in the biplot. It was found that By855, GY386B, and CML118 were distinct from the other genotypes from both locations because of their relatively high levels of vitamin B6 derivatives. Moreover, this analysis identified a close correlation between PLP and PMP, the major contributors to PC2. Close correlations were also observed between PL and PM, as well as between PM and PN (Figure 3C). Interestingly, in the case of the inbred-hybrid comparison, the biplot showed five separate and non-overlapping clusters for each derivative of vitamin B6. There were correlations among the derivatives of Vitamin B6 (between PLP and PMP, and PL and PM), and the major contributor to PC1 was PN, for PC 2 were PLP and PMP, respectively (Figure 3D).

### Impact of genotypes on vitamin variations

A two-way ANONA was conducted to see the genotype-environment interactions and their impact on the variation of vitamin B across the genotypes (Supplementary Table 3). The results indicated that genotypes have a significant impact on variations in each kind of vitamin B as well as the interaction of genotype-location is also significant. In Supplementary Table 4, the genotypes among which there is a significant difference in vitamins are presented in the presence of variable location and interaction. These findings revealed that even though there was a significant (*p* < 0.01) effect of the environment on the variation of the vitamin contents, the genetic basis was also exclusively and significantly (*p* < 0.01) responsible for the observed variations across the germplasms.

More precisely, to figure out if genotypes have an impact on vitamin variations, 30 inbred lines with the highest level of the vitamin were selected for further analysis. Not surprisingly, variation in each vitamin between the two locations was observed, indicating the existence of environmental effects on vitamin accumulation in the inbred lines. Our results also demonstrated that the variation among the lines grown in the same location can be largely attributed to their genotypes. Notably, some of these thirty lines were consistently high in one or more vitamins in both locations, including 9 lines for B1, 8 lines for B2, 12 lines for B3, 9 lines for B5 and 12 lines for B6 (Supplementary Figure 3). Besides, the inbred lines identified with high levels of multiple vitamins are CIMBL60 (B1 and B6), CIMBL154 (B1 and B3), Si273 (B1 and B6), GEMS33 (B2, B3, and B5), CML69 (B2 and B3), CIMBL96 (B2, B3 and B6), and By855 (B5 and B6). Moreover, some of these lines showed high levels of multiple B6 derivatives, including CML116 (PN and PM), M165 (PN and PM), CIMBL60 (PN, PL, and PM), By855 (PN, PL, PM, and PLP), GY386B (PN and PM), Gy386 (PN and PM), CML118 (PN, PL, and PM), CIMBL136 (PL and PMP), Gy1032 (PL and PLP), and Mo17 (PL and PMP).

## Discussion

Even though a broad range of vitamin B variations are observed among the germplasms in this study, most of them are far away from meeting the RDA (Supplementary Table 1). Fortunately, several lines are found to contain high levels of vitamins and hence can serve as promising sources to satisfy the RDA requirement if 100–300 g of kernels are consumed a day, such as CIMBL154 high in vitamin B1 (1.22 ± 0.10 mg/100 g) and By855 high in vitamin B6 (1.1 ± 0.06 mg/100 g) (Supplementary Table 2). It’s also been observed that the maize inbred lines differ remarkably in vitamin B accumulation when responding to different environmental cues. However, our findings revealed that several lines, By855 (B1, B2, B3, B5 and total B6, including PN, PL, PM, and PLP), Si273 (B1, B2, B3, B5, and total B6), GY386B (PN and PM) and CML118 (PN, PL, and PM) have been identified as rich sources of vitamin B, which were consistently high in vitamins in both locations. Thus, potentially, they can be used as promising candidate parent lines in hybrid breeding to enhance vitamin B in maize. Similarly, inbred of *Arabidopsis lyrata* plants [Vitamin B1, B2, and B6; (44)] and maize inbred lines [carotenoid in maize; (45, 46)] were also found to be less environmentally affected with respect to vitamin metabolism. These results highlight the importance of elucidating environmental effects on vitamin accumulation.

Different crops vary in the content and composition of vitamins. It has been found that oats (*Avena sativa*) and green peas (*Pisum sativum*) are rich in vitamin B1 (47). Our results confirm that maize grain is another suitable source of some vitamin B, such as vitamin B1 and B6. Moreover, both the heatmap and the violin plot showed a high degree of variation in thiamine among the maize lines (Figure 1 and Supplementary Figure 2), as has been found in quinoa [*Chenopodium quinoa*, (48)], cassava (13) and wheat (49). Pattern comparison of violin plots for locations showed environment had only a mild effect on the contents of PN and PMP (Supplementary Figures 2F,I). The variations and contents of vitamin B also vary among plant species, being higher in legumes in the case of thiamine and riboflavin than in cereal grains (50). Such variations of vitamin B1 and B2 have also been reported in wheat and wheat products in other studies, implying that they may be associated with genetic control and variety-dependent (50, 51). Studies found that, in the maize kernel, the content of vitamin B3 is low and substandard for human dietary requirements (52, 53). Our study indicated that maize grains from identified lines are a suitable source of vitamin B1, B2, B3, and B6, and dietary diversity will make up for other vitamin B in maize-based foods, similar to the conclusion of Palacios−Rojas et al. (54) that the high diversity of maize is a critical factor to improve nutritional profiles in humans through biofortification.

The possible interactions among vitamins in plant metabolism have been observed at different levels. For example, several studies have reported that thiamine and pyridoxine interplay and influence different metabolic pathways (43). In the present study, we also observed that not only thiamine (vitamin B1) but also pantothenic acid (vitamin B5) were positively associated with total vitamin B6 (Figures 2A,B). Thus, it’s important to take into consideration the potential vitamin-vitamin interaction when the inbred lines are used as parental lines. There is a correlation between PN and PM as well as between PL and PM (Figures 2D–F, 3C,D), which can be explained by cytosolic interconversions of these derivatives in plant cells (43). Interestingly, riboflavin was found to have no correlation with other vitamin B in this study, which is similarly a proven biochemical finding in wheat by Shewry et al. (49). A comprehensive study of these interactions could help enhance a certain vitamin in maize. Moreover, we also found that the contents of vitamin B1 and B6 were much lower in the hybrids than in the inbred lines in our study (Table 2), indicating a necessity to improve the hybrids for better nutrition quality. Taking this into consideration, both parental lines may need to be simultaneously genetically modified to achieve vitamin accumulation as high as possible.

Vitamin B, specifically vitamers, and their homeostasis within the tissue, organ, and subcellular levels are essential for the improved health of both humans and plants (55, 56). Not only as human food, but also as internal metabolites in plants, they play major physiological roles that include disease resistance [vitamin B6, tomato and *Arabidopsis thaliana*, (57)], a protective role against UV-B supporting peroxidase defense linked to the biosynthesis of PLP [*Arabidopsis thaliana*, (58)], tolerance to salinity (NaCl) or methyl viologen-induced stress [vitamin B6, potato, (59)] and drought [vitamin B3, *Arabidopsis thaliana*, (60)]. Thus, our Vitamin B study on maize could further facilitate the research on plant stress metabolism and can help develop stress-tolerant varieties.

Nutrition enrichment in plants, especially in grain crops, is one of the most challenging tasks to improve the nutritional status of the population in the scenario of global climate change. Researchers have already applied several strategies and tools for the development of nutritious cereals to accelerate the reduction of malnutrition in humans and improve our well-being (61, 62). Among them, biofortification in crops has been found to be successful in recent years through agronomic practices (63), metabolic engineering (64, 65) and genome-assisted breeding (66), such as rice endosperm for riboflavin (67) and thiamin (68), and selenium biofortification in maize (69), to mitigate malnutrition among the world population (70). Biofortification in maize could be a practical solution to increase vitamin intake sustainably (10, 71). Successful strategies for improving maize nutritional quality, particularly biofortification, were critical in reducing malnutrition and improving nutritional health status among their populations in Eastern and Southern Africa where micronutrient deficiencies were prominent (72). In such cases, germplasm resources with a considerable level of vitamin B can be appropriate for micronutrient enhancement breeding initiatives in maize through molecular mechanisms and functional genomics research (73, 74). Marker-assisted selection for cost-effective breeding and quantitative trait loci studies for candidate gene identification (75), the turn-over activity of genes, and targeted gene editing or metabolic engineering have already been proposed as effective approaches for thiamine modification (68, 76, 77). So is in maize niacin improvement (78). In such future research, our elite inbred could be a potential germplasm resource. Notably, vitamin B6 has also attained global concern in crop biofortification in recent years for the betterment of human health (79). Biofortification has also gained a greater interest to provide desired and adequate nutritious feed and fodder to poultry birds, animals for the production of meat and dairy products (80), while the ultimate goal is to reduce hidden hunger.

## Conclusion

Vitamin B is involved in various biological processes of cell functioning, energy metabolism, and the immune system through numerous pathways as cofactors, precursors, and substrates, in both plants and humans (81). Humans rely on the dietary uptake of vitamin B, directly or indirectly, from plants, microbes, and animals. Among the dietary sources, maize, which is consumed as a staple food in several low and middle-income countries, has become an integrated part of their tradition and culture (55). Despite this, two billion people suffer from micronutrient deficiencies and have limited access to nutritious foods. Thus, biofortified maize containing vitamin B and other essential micronutrients (such as vitamin A and vitamin E) could play a significant role in improving nutrient deficiencies among the population having limited dietary diversity. In our study, a wide range of variations of vitamin B was observed among the inbred germplasms between two locations, which has made this study the first detailed report to see the role of genotype and environmental interactions on these vitamins. Moreover, the content of individual vitamin B in several germplasms has surpassed the RDA requirement of the human diet. The interactions and correlations between B1, B5, and B6, as well as between PN and PM, have opened a route for more detailed molecular studies of vitamin B homeostatic regulation in plants and grain. This study found that the contents of these vitamins differed between Hainan and Hebei, but this could be due to genetics, as several inbred lines showed consistently high measured values. It is also interesting to see that hybrids compared to inbreds have a lower content of vitamin B, which suggests that the hybrids need to be improved in nutritional quality. One of the ways it can be obtained is by deploying these inbred lines containing higher quantities of vitamin B and their introgression into the elite hybrids containing other vitamins like A and E. Our study broadens the scope of selecting inbred lines with diverse profiles and has identified suitable maize germplasm for further breeding programs for biofortification, thus improving the nutritional quality of diets.

## Data availability statement

The data presented in the study is available in the Supplementary material.

## Author contributions

FS, TL, and LJ analyzed the data and wrote the manuscript. LJ and CZ designed the study. QL and WW collected maize inbred lines and grew them. LJ, TL, FS, and QL performed the analysis of vitamin B. CZ supervised the project and revised the manuscript. All authors have read and approved the final manuscript.

## Footnotes

1
www.sigmaaldrich.com

2
www.solarbio.com

3
www.merckmillipore.com

4
www.spexsampleprep.com

5
www.tosoh.co.jp

6
www.waters.com

7
www.sciex.com.cn

## Supplementary material

Supplementary Figure 1 Variation of vitamin B contents in maize inbred lines from sub-groups in boxplots. Axes: x, sub-groups; y, vitamin content (μg/100 g). In each plot, straight line, box and white rectangular point represented the range, interquartile range and the median of the data, respectively. Legends: B1, thiamine; B2, riboflavin; B3, niacinamide; B5, pantothenic acid; and B6, total vitamin B6.

Supplementary Figure 2 Distribution patterns of germplasms for contents of vitamin B1, B2, B3, B5, total B6, and vitamers of B6. Axes: x, Group; y, Vitamin content (μg/100 g). Legends: **(A)** B1, thiamine; **(B)** B2, riboflavin; **(C)** B3, niacinamide; **(D)** B5, pantothenic acid; and **(E)** B6, total of vitamin B6 and derivatives; **(F)** PL, pyridoxal; **(G)** PLP, pyridoxal 5’-phosphate; **(H)** PM, pyridoxamine; **(I)** PMP, pyridoxamine-5’-phosphate; **(J)** PN, pyridoxine.

Supplementary Figure 3 Variation in content of each vitamin B from selected germplasms that ranked high in both locations. Values are presented as means ± SD. x-axis: Content, y-axis: Germplasm name. Panels: Tornado graphs. **(A)** B1, thiamine; **(B)** B2; riboflavin; **(C)** B6, pyridoxine; **(D)** B5, pantothenic acid; **(E)** B3, niacinamide; **(F)** PLP, pyridoxal 5’-phosphate; **(G)** PN, pyridoxine; **(H)** PM, pyridoxamine; **(I)** PMP, pyridoxamine-5’-phosphate; **(J)** PL, pyridoxal.

Supplementary Table 2 The contents (μg/100 g) of each vitamin B in grains of each inbred line grown in Hainan and Hebei province and commercial hybrid lines grown in Hebei.

Supplementary Table 4 Pairwise comparison for vitamins across genotypes from two-way ANOVA.
