# Supplementary material for: Variation of vitamin B contents in maize inbred lines: Potential genetic resources for biofortification
Source: Front Nutr. 2022 Oct 21;9:1029119. doi: 10.3389/fnut.2022.1029119 (PMC9634661; doi:10.3389/fnut.2022.1029119)
Supplement: Supplementary file 1 [file Data_Sheet_1.zip › Supplementary Table 1.docx]

Supplementary Table 1: Overall ranges of vitamin B contents among the inbred lines of maize

| **Compound** | **Content** (μg/100g) |
| --- | --- |
| Vitamin B1 | 107.61 ± 16.26 to 2654.54 ± 113.7 |
| Vitamin B2 | 1.19 ± 0.19 to 37.37 ± 2.44 |
| Vitamin B3 | 19.6 ± 0.39 to 213.75 ± 17.68 |
| Vitamin B5 | 43.47 ± 5.42 to 590.86 ± 4.18 |
| Total vitamin B6 | 138.59 ± 12.08 to 1065.11 ± 56.93 |
