# Supplementary material for: Variation of vitamin B contents in maize inbred lines: Potential genetic resources for biofortification
Source: Front Nutr. 2022 Oct 21;9:1029119. doi: 10.3389/fnut.2022.1029119 (PMC9634661; doi:10.3389/fnut.2022.1029119)
Supplement: Supplementary file 1 [file Data_Sheet_1.zip › Supplementary Table 3.docx]

**Supplementary Table 3**: Two-way ANOVA of the studied genotypes in environmental locations*

| **Vitamin** | **Source of variation** | **Sum of Squares** | **Mean Square** | **F value** | **p-value** | **SS%** |
| --- | --- | --- | --- | --- | --- | --- |
| **B1** | Genotype(G) | 46650280 | 300970 | 82.08 | <0.01 | 52.1358499 |
|  | Location(L) | 10750325 | 10750325 | 2932.01 | <0.01 | 12.0144473 |
|  | G × L | 30933748 | 199573 | 54.43 | <0.01 | 34.57122321 |
|  | Error | 1143962 | 3667 |  |  |  |
|  | Total | 89478315 |  |  |  |  |
| **B2** | Genotype(G) | 9197 | 59 | 38.25 | <0.01 | 25.40327036 |
|  | Location(L) | 21104 | 21104 | 13603.24 | <0.01 | 58.29190145 |
|  | G × L | 5419 | 35 | 22.54 | <0.01 | 14.96795934 |
|  | Error | 484 | 2 |  |  |  |
|  | Total | 36204 |  |  |  |  |
| **B3** | Genotype(G) | 235140 | 1517 | 23.79 | <0.01 | 21.02191504 |
|  | Location(L) | 724240 | 724240 | 11357.54 | <0.01 | 64.74828505 |
|  | G × L | 139272 | 899 | 14.09 | <0.01 | 12.45115315 |
|  | Error | 19895 | 64 |  |  |  |
|  | Total | 1118547 |  |  |  |  |
| **B5** | Genotype(G) | 2847040 | 18368 | 43.28 | <0.01 | 53.17706556 |
|  | Location(L) | 579391 | 579391 | 1365.31 | <0.01 | 10.82187577 |
|  | G × L | 1795054 | 11581 | 27.29 | <0.01 | 33.52805168 |
|  | Error | 132402 | 424 |  |  |  |
|  | Total | 5353887 |  |  |  |  |
| **B6** | Genotype(G) | 258187 | 1666 | 120.12 | <0.01 | 67.00064876 |
|  | Location(L) | 11597 | 11597 | 836.28 | <0.01 | 3.009471909 |
|  | G × L | 111239 | 718 | 51.75 | <0.01 | 28.86700402 |
|  | Error | 4327 | 14 |  |  |  |
|  | Total | 385350 |  |  |  |  |

*For traits measured on individual vitamin: df of genotype = 155, df of location = 1, df of G × L = 155, df of Error = 312, df of total = 623
