# Supplementary figures and images for: Variation of vitamin B contents in maize inbred lines: Potential genetic resources for biofortification
Source: Front Nutr. 2022 Oct 21;9:1029119. doi: 10.3389/fnut.2022.1029119 (PMC9634661; doi:10.3389/fnut.2022.1029119)

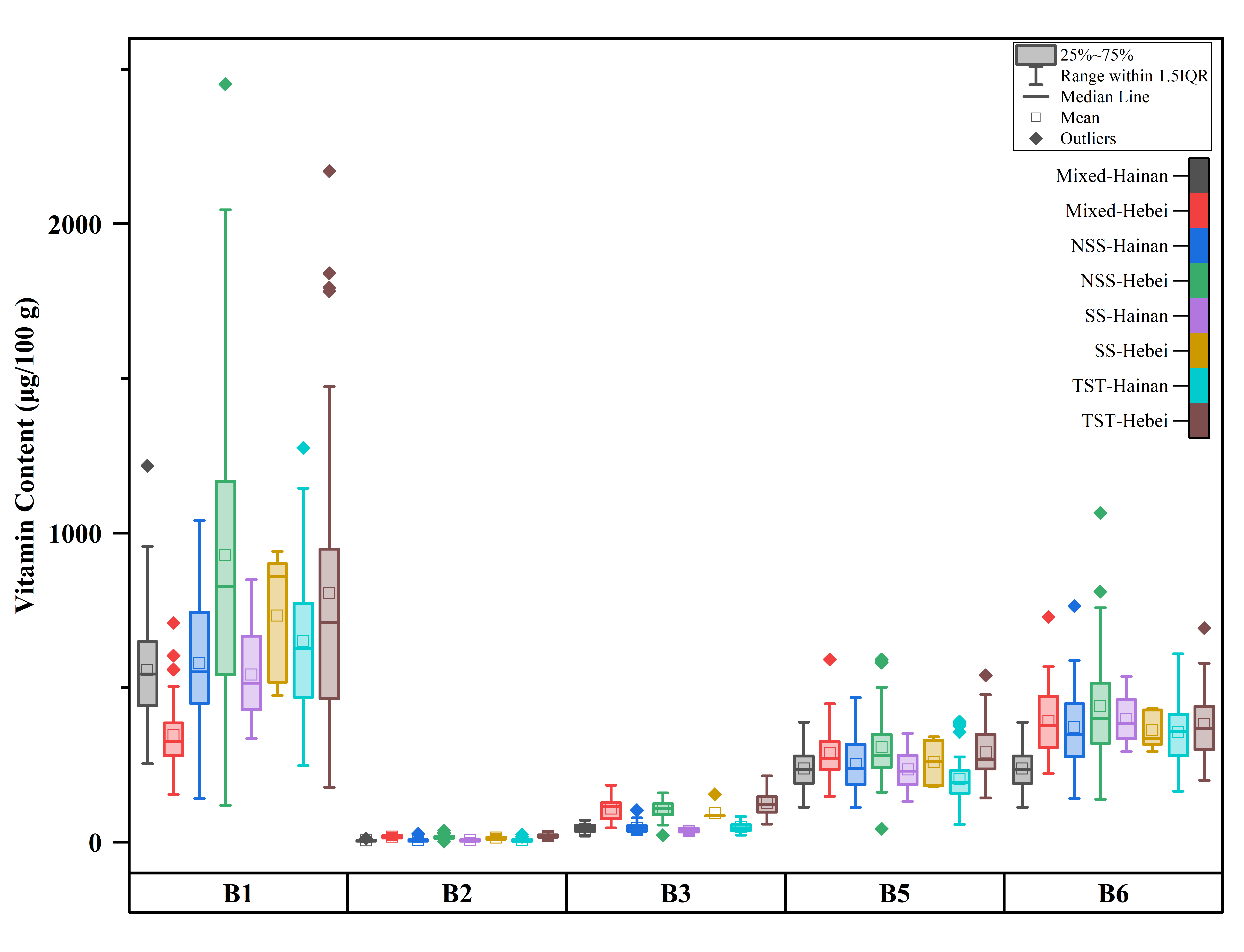

Supplement: Supplementary Figure 1 — Variation of vitamin B contents in maize inbred lines from sub-groups in boxplots. Axes: x, sub-groups; y, vitamin content (μg/100 g). In each plot, straight line, box and white rectangular point represented the range, interquartile range and the median of the data, respectively. Legends: B1, thiamine; B2, riboflavin; B3, niacinamide; B5, pantothenic acid; and B6, total vitamin B6. [file Image_1.jpg]

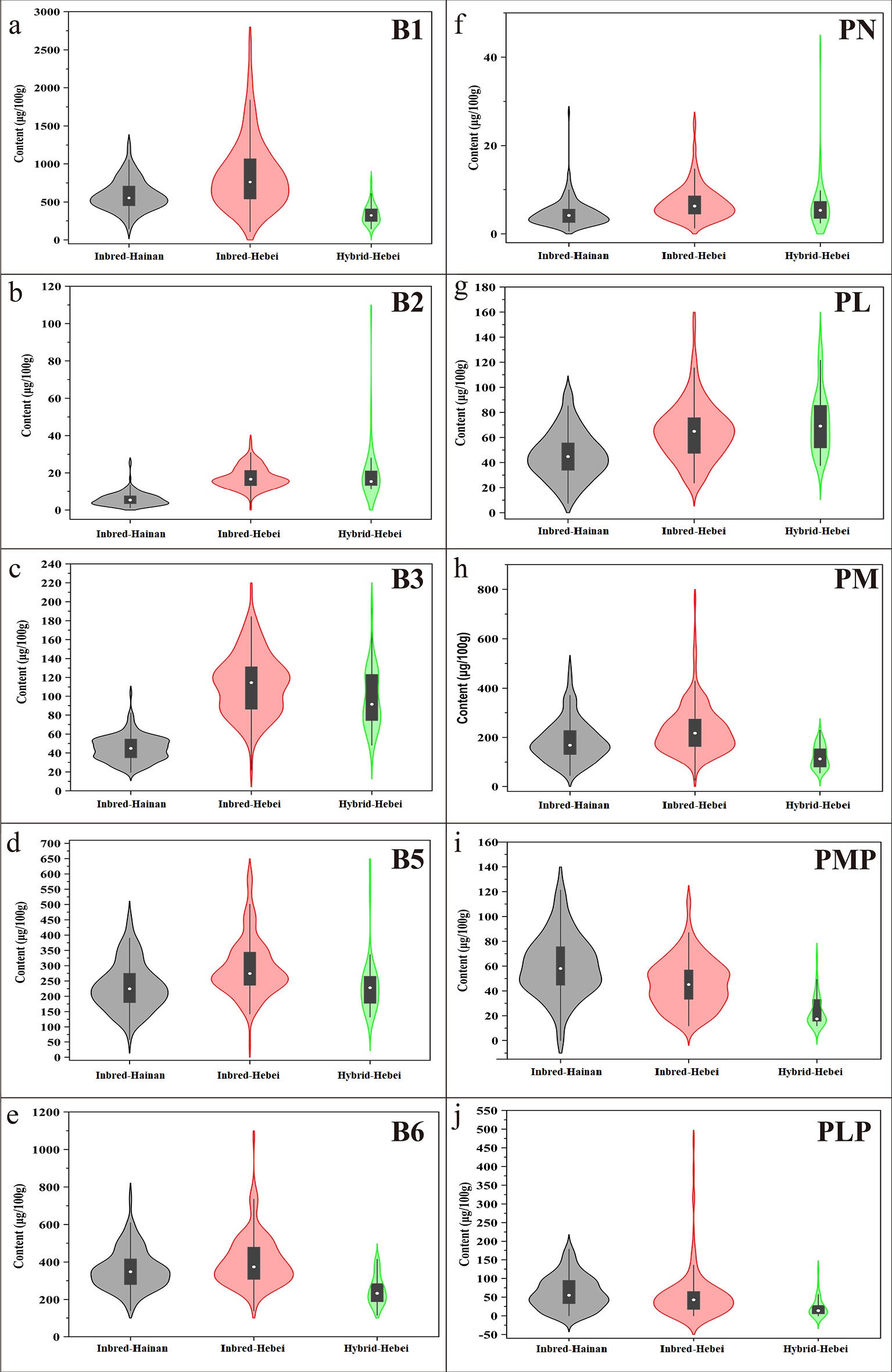

Supplement: Supplementary Figure 2 — Distribution patterns of germplasms for contents of vitamin B1, B2, B3, B5, total B6, and vitamers of B6. Axes: x, Group; y, Vitamin content (μg/100 g). Legends: (A) B1, thiamine; (B) B2, riboflavin; (C) B3, niacinamide; (D) B5, pantothenic acid; and (E) B6, total of vitamin B6 and derivatives; (F) PL, pyridoxal; (G) PLP, pyridoxal 5’-phosphate; (H) PM, pyridoxamine; (I) PMP, pyridoxamine-5’-phosphate; (J) PN, pyridoxine. [file Image_2.jpg]

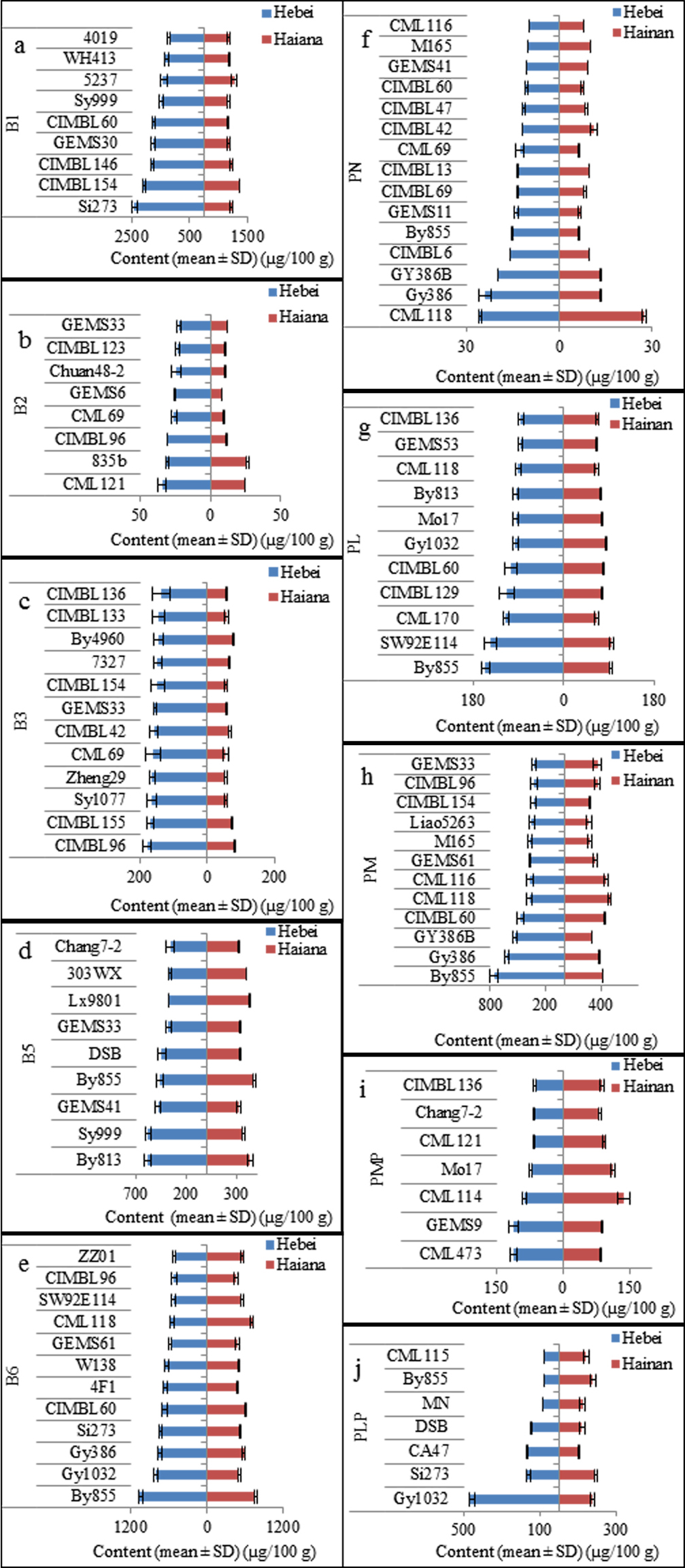

Supplement: Supplementary Figure 3 — Variation in content of each vitamin B from selected germplasms that ranked high in both locations. Values are presented as means ± SD. x-axis: Content, y-axis: Germplasm name. Panels: Tornado graphs. (A) B1, thiamine; (B) B2; riboflavin; (C) B6, pyridoxine; (D) B5, pantothenic acid; (E) B3, niacinamide; (F) PLP, pyridoxal 5’-phosphate; (G) PN, pyridoxine; (H) PM, pyridoxamine; (I) PMP, pyridoxamine-5’-phosphate; (J) PL, pyridoxal. [file Image_3.jpg]
